# Systematic Nutritional Clinical Assessment (SyNCA): Instrument Development, Delphi Protocol for Content, and Semantic Validation

**DOI:** 10.1111/jhn.70235

**Published:** 2026-03-24

**Authors:** Rosangela Passos de Jesus, Lucivalda Pereira Magalhães de Oliveira, Carla de Magalhães Cunha, Ramona Souza da Silva Baqueiro Boulhosa, Ana Carolina Braga Porto Ricci, Monalisa Reis Arruda, Ana Clara Vital Batista, Ketsia Meneses Souza Santos, Ketlin Gianini Pereira, Fernando Gomes Romeiro, Andre de Castro Lyra, Allain Amador Bueno

**Affiliations:** ^1^ Graduate Program in Food, Nutrition, and Health, School of Nutrition Federal University of Bahia Salvador Bahia Brazil; ^2^ Departament of Nutrition Midwest State University Guarapuava Paraná Brazil; ^3^ Graduate Program in Pathophysiology in Internal Medicine, Botucatu Medical School São Paulo State University Avenida Professor Montenegro, s/n, Vila Paraíso Botucatu São Paulo Brazil; ^4^ Department of School Nutrition Municipal Department of Education Paulínia São Paulo Brazil; ^5^ School of Medicine Federal University of Bahia Largo Terreiro de Jesus, s/n, Pelourinho Salvador Bahia Brazil; ^6^ College of Health, Life and Environmental Sciences University of Worcester Worcester UK

**Keywords:** chronic diseases, malnutrition, nutritional assessment, nutrition‐focused physical examination, subjective methods

## Abstract

**Background:**

Malnutrition is a common complication in patients with chronic diseases, often exacerbated by clinical factors and increased metabolic demands. In individuals affected by Decompensated Chronic Liver Disease (DCLD), fluid retention, such as oedema and ascites, can hinder accurate nutritional assessment, leading to underdiagnosis or misdiagnosis of weight loss and malnutrition. The present study describes the development and validation of a novel tool for Systematic Nutritional Clinical Assessment (SyNCA), including its content, semantic, and construct validity.

**Methods:**

In Phase 1, the Delphi protocol was employed to evaluate SyNCA content and semantic validity with input from clinical nutrition experts and resident nutritionists. Phase 2 involved a cross‐sectional, multicentre study of DCLD hospitalised patients to assess construct validity. SyNCA outcomes were compared with established nutritional assessment methods including anthropometry, handgrip strength (HGS), and dual‐energy X‐ray absorptiometry (DEXA). Correlations were analysed using Pearson or Spearman coefficients.

**Results:**

Following expert review in Phase 1, out of the 18 items initially proposed across five anatomical regions, 14 items were retained in the final SyNCA instrument. In Phase 2, data from 136 hospitalised DCLD patients revealed moderate correlations between SyNCA scores and arm muscle circumference (*r* = −0.567, *p* < 0.0001), HGS (*r* = −0.376, *p* < 0.0001), and Appendicular Muscle Mass Index (*r* = −0.502, *p* < 0.001), supporting construct validity.

**Conclusion:**

SyNCA demonstrated strong content, semantic, and construct validity, demonstrating its potential as a reliable clinical tool for nutritional assessment in DCLD hospitalised patients, and particularly welcomed in clinical settings with limited resources or where traditional methods are impractical.

## Summary

- The presence of oedema and ascites impairs nutritional assessment due to fluid retention, leading to the underdiagnosis of malnutrition and weight loss in affected patients.
- We developed and undertook content and semantic validation using the Delphi protocol (Phase 1), and construct validation (Phase 2), of a low‐cost instrument for Systematic Nutritional Clinical Assessment (SyNCA).
- Phase 2 was a multicentre study involving 136 patients with decompensated chronic liver disease, assessed using SyNCA, anthropometry, Handgrip Strength (HGS), and Dual‐Energy X‐ray Absorptiometry.
- SyNCA demonstrated strong content and semantic validity, alongside moderate correlations between its scores and mid‐upper arm muscle circumference, HGS, and Appendicular Skeletal Muscle Mass Index.
- SyNCA may serve as a reliable clinical tool for nutritional assessment of hospital inpatients, particularly in resource‐limited settings or where traditional objective methods are unfeasible or prohibitively costly.

## 1 Introduction

Patients with chronic diseases frequently experience metabolic and nutritional alterations that negatively impact clinical outcomes and quality of life, with hospitalised individuals being particularly at risk [1, 2]. Malnutrition, for example, is strongly linked to increased rates of morbidity and mortality [3, 4]. Key contributors to malnutrition in patients affected by chronic conditions include complications related to the underlying disease, ageing, progressive loss of functional and cognitive capacity, hospital‐related factors such as unpalatable meals and restrictive diets, and unhealthy lifestyle habits [5, 6].

Despite the high prevalence of malnutrition, particularly among older adults, in patients undergoing invasive treatments, and in those with comorbidities, it is frequently misdiagnosed or underdiagnosed in hospital settings. This is largely explained by a lack of reliable diagnostic tools and limited availability of objective assessment methods [1]. Studies suggest that the prevalence of malnutrition among hospitalised patients can range from 5% to 92%, depending on the assessment method employed [1, 7, 8].

To address this issue, the American Society for Parenteral and Enteral Nutrition (ASPEN) Working Group on Malnutrition has recommended the use of subjective assessment methods, such as the Nutrition‐Focused Physical Examination (NFPE), which helps identify clinical signs of subcutaneous fat loss, muscle wasting, fluid retention, and micronutrient deficiencies [9, 10]. Subjective methods, including the NFPE, are particularly valuable in patients affected by fluid retention, such as oedema or ascites, where objective methods may be less accurate [9, 11].

Despite the NFPE being a non‐invasive tool employed in some clinical settings, there remains a lack of standardised protocols and validated instruments for conducting comprehensive nutritional assessments. This deficiency is particularly relevant in public hospital settings, which are often characterised by excess patient numbers, poor infrastructure, underfunding, and understaffing.

Recognising the need for an efficient and inexpensive method of nutritional clinical assessment with high reliability and reproducibility, the present study aimed to firstly describe the process of developing and refining the content and semantics of a new nutritional assessment tool, and secondly, to validate the construction of the “Systematic Nutritional Clinical Assessment” (SyNCA) instrument for clinical use.

## 2 Methods

### 2.1 Study Design

This was a multicentre observational study conducted in two phases. In Phase 1, the SyNCA instrument was developed and subjected to content and semantic validation. In Phase 2, a multicentre cross‐sectional study was carried out to assess the instrument's validity in clinical practice. The study received approval from the Research Ethics Committees of the three participating hospitals in Brazil, namely Professor Edgard Santos University Hospital Complex (HUPES, Bahia Federal University, Salvador), Bahia State Roberto Santos General Hospital (HGRS, Salvador), and Clinical Hospital of the Medical Faculty of Botucatu (HCFMB). All participants in both phases voluntarily agreed to take part and signed a standardised Free and Informed Consent Form.

### 2.2 Phase 1: Development, Validation of Content and Semantic, Delphi Protocol

#### 2.2.1 Development

The first SyNCA version was created by a team of three University nutrition professors (RPJ, CMC, LPMO) based on existing literature and their clinical expertise. After several rounds of discussion and refinement, a preliminary version of the SyNCA instrument was finalised. The proposed structure involved a longitudinal assessment of the patient, starting from the cranial region down to the feet, covering five anatomical areas: face, anterior trunk, posterior trunk, upper limbs, and lower limbs.

The physical examination guided by the SyNCA is structured around three core principles: detailed description of each body segment assessed, standardised technique and patient positioning for inspection and palpation, and a scoring system by body segment, culminating in an overall evaluation result. Techniques and positioning were illustrated (Supplementary Table 1, SyNCA Instrument), alongside detailed descriptions of the necessary manoeuvres to assess the degree of tissue depletion in specific anatomical areas.

The sum of the individual scores yields the Nutritional Depletion Index (NDI), which provides a composite indicator of the patient's nutritional status. The final score is stratified into quartiles. For each body region, a pre‐established scoring system based on the observed degree of tissue depletion is applied, defining four diagnostic levels: 0 = no signs of depletion; 2 = mild depletion; 4 = moderate depletion; and 6 = severe depletion.

### 2.3 Content and Semantic Validation

The SyNCA content validation was conducted from May to December 2021 using the Delphi protocol [12, 13, 14]. Invitations to collaborate on the study were extended to 50 experts drawn from the register of clinical nutrition specialists engaged in scientific activities within the Brazilian Nutrition Association and the Brazilian Society of Parenteral and Enteral Nutrition, ensuring national representation. Of these, 18 initially agreed to contribute, and 17 returned the completed survey. Their names are presented at the end of this document. Literature suggests that a minimum of 15 experts is required for robust content validation using the Delphi protocol [13, 14].

Both content and semantic validation phases were conducted via an online questionnaire (Google Forms®), sent to the invited clinical nutrition experts, alongside a consent form. All panel members, who were state‐registered nutritionists in Brazil with postgraduate clinical credentials and recognised clinical expertise, agreed to participate in the study. In addition to rating the instrument, experts were asked to offer comments, identify potential weaknesses, and suggest improvements to the proposed instrument.

In the first round of evaluation concerning the attributes of the body segments, the panel of clinical nutrition experts was asked to evaluate whether the anatomical regions included were essential for nutritional assessment, and to comment on the adequacy of the described techniques and illustrations for the identification of tissue depletion through visual inspection and palpation. Participants could not see other participants' comments and scores.

A 5‐point Likert‐type scale was used for this evaluation: 1—not relevant/pertinent/representative/understandable; 2—somewhat relevant/pertinent/representative/understandable; 3—not sure; 4—relevant/pertinent/representative/understandable; 5—very relevant/pertinent/representative/understandable. The Content Validity Index (CVI) was calculated as the proportion of experts assigning scores of 4 or 5 to each item. A CVI ≥ 0.80 was considered indicative of consensus, in line with Davis (1992) [15]. Items with a CVI below 0.80 were revised, restructured whenever possible, and included in a second round of evaluation. In this subsequent round, participants were permitted to revise the scores they had assigned in the first round for items which had not achieved consensus. A single, additional round was conducted to evaluate the attributes pertaining to the multiple‐choice answer options for each body segment (Table 1).

**Table 1:** Evaluation of Anatomical Regions, Body Segments, and Multiple‐choice Answer Options Proposed for the Systematic Nutritional Clinical Assessment (SyNCA) Instrument, and Changes After Content Validation for the Final Version of the Proposed Instrument.

Domains: Anatomical regions	Item	Body segments	Body segments Attributes: Relevance, pertinence, representativeness		Multiple‐choice answer options for each body segment.
					Attributes: Relevance, pertinence, clarity
			1st cycle – 18 clinical experts	2nd cycle – 17 clinical experts	1st cycle – 17 clinical experts
1. Face CVI ≥ 0.80 for all items	1	Orbital region	Failed in all 3 attributes	_	_
			CVI = 0.72 Removed		
	2	Temporal (temporalis muscle)	CVI > 0.80 Approved	_	CVI = 1.0 Approved
	3	Masseter (chewing muscle)	CVI ≥ 0.89 Approved	_	CVI ≥ 0.94 Approved
	4	Bichat's fat pad (buccal fat pad)	CVI ≥ 0.89 Approved	_	CVI ≥ 0.94 Approved
	5	Zygomatic arch & mandible lines	CVI = 0.94 Approved	_	CVI ≥ 0.88 Approved
2. Trunk Frontal region CVI = 1.0 for all items	6	Trapezius and Pectoralis Major (muscles covering shoulder, clavicle, and chest)	CVI = 0.94 Approved	_	CVI ≥ 0.88 Approved
	7	Intercostal muscles (muscles covering ribs and around sternum)	CVI ≥ 0.88 Approved	_	CVI ≥ 0.82 Approved
3. Trunk Dorsal region CVI = 0.77 for pertinence CVI ≥ 0.80 for other items	8	Subscapularis and Latissimus dorsi (muscles covering scapula and dorsal region)	No consensus among experts Relevance: CVI > 0.80	Failed representativeness CVI = 0.70 Removed	_
			Pertinence and Representativeness CVI ≤ 0.78		
			Reassessed in 2nd cycle		
	9	Paravertebral muscles (muscles supporting spinal column)	No consensus among experts Relevance: CVI > 0.89	Failed pertinence and representativeness CVI ≤ 0.58 Removed	_
			Pertinence and Representativeness CVI ≤ 0.78		
			Reassessed in 2nd cycle		
	10	Deltoid muscle (muscle covering acromion, acromial angle of the scapula, and upper humerus)	Approved in all three attributes CVI ≥ 0.83	_	CVI ≥ 0.82 Approved
4. Upper limbs CVI ≥ 0.80 for all items	11	Biceps and Triceps (arm muscles)	CVI ≥ 0.89 Approved	_	CVI ≥ 0.94 Approved
	12	Adductor muscle of the thumb	CVI = 0.89 Approved	_	CVI ≥ 0.88 Approved
	13	Dorsum of the hands, interosseous muscles	CVI = 0.83 Approved	_	CVI ≥ 0.82 Approved
5. Lower limbs CVI ≥ 0.80 for all items	14	Quadriceps (muscles located on the anterior thigh)	Failed in pertinence CVI = 0.78 Approved for relevance and representativeness CVI = 0.89	Reassessed, failed in pertinence: CVI = 0.76	CVI ≥ 0,94 Approved
				Approved for representativeness CVI = 0.89	
				Maintained by team decision	
	15	Gastrocnemius (back of leg below knees)	CVI ≥ 0.94 Approved	_	CVI ≥ 0.82 Approved
	16	General inspection of the lower limb bone structures: hips, legs, knees and feet	Failed in pertinence CVI = 0.78 Approved for relevance and representativeness CVI ≥ 0.83	Reassessed, approved for pertinence CVI > 0.82 Maintained	CVI ≥ 0.88 Approved
	17	Feet and ankles – Malleolar region	Failed in all three attributes	CVI ≤ 0.72 Removed	_
	18	Presence of cutaneous oedema in the lower limbs	CVI ≥ 0.89 Approved	_	CVI ≥ 0.94 Approved

*Note:* Acceptable CVI ≥ 0.80.

*Abbreviations*: CVI, Content Validity Index.

Following completion of the content validation stage, the instrument underwent semantic validation. This phase aimed to assess the clarity and comprehensibility of both the items and the scoring system. For this semantic validation process, 14 nutritionists were invited to participate. These individuals were in their second year of the Clinical Nutrition Residency Programme at the School of Nutrition, Federal University of Bahia, and possessed relevant experience in performing the activities for which the proposed instrument is intended.

All professionals were individually invited via intranet email to participate voluntarily in the present study. Those who agreed to take part received a consent form along with the first version of the instrument and instructions on how to evaluate it, and any requested clarifications were provided prior to their contribution to the study.

The semantic validation questionnaire used the following 5‐point Likert‐type scale: 1—not understandable; 2—somewhat understandable; 3—not sure; 4—understandable; 5—very understandable. Items with a CVI ≥ 0.80 were considered clear and acceptable. The SyNCA final version was established after critical analysis of the qualitative feedback and quantitative results by the research team.

**Figure 1:**
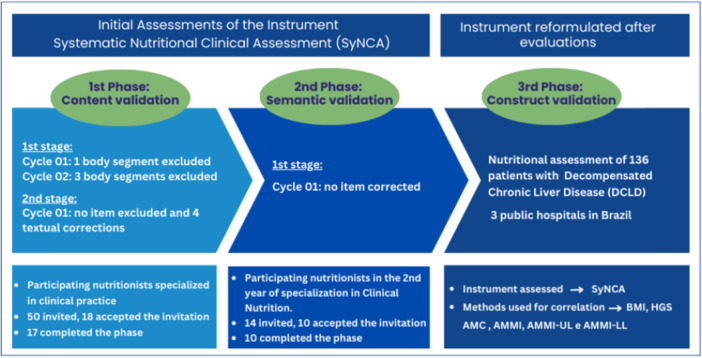
Instrument development process, participant selection, and characterisation of the content and semantic validation by the clinician experts. Legend: DCLD, Decompensated Chronic Liver Disease. Synca, Systematic Nutritional Clinical Assessment. BMI, Body Mass Index. HGS, Handgrip Strength. AMC, Arm Muscle Circumference. AMMI, Appendicular Muscle Mass Index. AMMI‐UL, upper limb AMMI. AMMI‐LL, lower limb AMMI.

Figure 1 provides an overview of the instrument development process, participant selection, activity sequence, and characterisation of the content and semantic validation by the clinician experts.

### 2.4 Phase 2: Construct Validity Assessment

The second phase of the study evaluated the SyNCA construct validity through nutritional assessments of 136 adult patients hospitalised for DCLD across three university hospitals in Brazil. The nutritionists from the research team and the hepatologist consultants working in the wards and outpatient clinics of the participating hospitals were responsible for identifying eligible patients for the study and extending the invitation to participate. Patient distribution was as follows: 59 were admitted to the Professor Edgard Santos University Hospital Complex (HUPES, Bahia Federal University, Salvador); 27 at the Bahia State Roberto Santos General Hospital (HGRS, Salvador); and 50 at the Clinical Hospital of the Medical Faculty of Botucatu (HCFMB).

### 2.5 Inclusion and Non‐Inclusion Criteria

Inclusion and non‐inclusion criteria were consistent across all three centres. Eligible participants were aged 18 years or older, with a previously established diagnosis of DCLD confirmed by a hepatologist consultant, hospitalised for DCLD treatment in a non‐critical condition, and capable of providing informed consent. Non‐inclusion criteria were set for patients with a diagnosis of heart disease, rheumatologic conditions, acquired immunodeficiency syndrome, active pulmonary tuberculosis, confirmed diagnosis of cancer, or any condition requiring isolation. A total of 136 patients met the inclusion criteria and consented to participate, exceeding the minimum recommended sample size of 100 for construct validation [16].

### 2.6 Data Collection and Nutritional Assessment Protocol

All nutritional assessments were conducted within the first 48 h of hospital admission and at the initial consultation for outpatient participants. Clinical data, including liver disease aetiology and severity, were obtained from electronic medical records.

### 2.7 Muscle and Fat Mass Evaluation

Arm Circumference (AC) was measured using a non‐elastic tape, and Triceps Skinfold Thickness (TSF) was measured using calibrated Lange® skinfold calipers [17, 18]. Arm Muscle Circumference (AMC) was calculated using the formula proposed by Blackburn and Thornton (1979) [19] using AC and TSF, with AMC adequacy (%) classified based on the 50th percentile for sex and age. An AMC adequacy of ≤ 90% was considered indicative of malnutrition.

### 2.8 Functional Assessment

Handgrip Strength (HGS) was assessed on the non‐dominant hand using a Jamar® dynamometer. Measurements were taken with patients seated, elbow flexed at 90°. Verbal instructions were provided, but no encouragement was provided during the measurements. Three readings were recorded, each separated by a 30‐second rest interval, with the dynamometer set to the second handle position [20, 21, 22, 23]. Average HGS values below 26 kilograms (Kg) for men and 18 kg for women were classified as reduced [24].

### 2.9 Reference Standard Assessment – DEXA

A subset of 47 patients underwent body composition analysis using Dual‐Energy X‐ray Absorptiometry (DEXA) at the HCFMB Hospital, employing a Lunar DPX NT densitometer (GE Healthcare®), set to a maximum capacity of 136 kg and height limit of 1.95 m. The Appendicular Muscle Mass Index (AMMI) was calculated as the sum of muscle mass from all four limbs in kilograms divided by the square of the patient's height in metres, following established methodology [25].

To support anatomical region‐specific validation of the SyNCA instrument, AMMI values for upper and lower limbs, extracted separately via DEXA, were compared with the SyNCA scores for the corresponding regions. For upper limbs, comparisons included the deltoid, biceps, triceps, adductor pollicis, and dorsum of the hands. For lower limbs, quadriceps, gastrocnemius, bone structures, and the presence of oedema were assessed.

### 2.10 Statistical Analysis

#### 2.10.1 Phase 1 (Content Validation and Semantic Analysis)

Statistical analysis was conducted using the CVI, which was calculated as the proportion of expert responses rated 4 or 5 on a 5‐point Likert scale relative to the total number of responses. The CVI reflects the degree of expert consensus regarding each item. Items with a CVI ≥ 0.80 were considered to have reached acceptable agreement [15].

To further assess inter‐rater agreement, the Fleiss' Kappa coefficient (fK) was calculated. This statistical tool adjusts for agreement expected at random and categorises the strength of agreement as follows: no agreement (0.00–0.19), weak (0.20–0.39), moderate (0.40–0.59), substantial (0.60–0.79), and almost perfect (0.80–1.00) agreement [26]. A fK ≥ 0.80 was accepted by the research team. Both CVI and fK were computed for each anatomical region, item, and choice of answer option, focusing on the evaluation of the instrument's relevance, pertinence, representativeness, clarity, and comprehensibility.

#### 2.10.2 Phase 2 (Construct Validity)

Qualitative anthropometric variables were reported as proportions, while quantitative variables were summarised using means and standard deviations, or medians and interquartile ranges, as appropriate, depending on the outcomes of the distribution normality tests. Group comparisons by sex were made using either Student's *t*‐test or Mann–Whitney *U* test. Construct validity was assessed via hypothesis testing, as described previously [27]. Correlations between final SyNCA scores and anthropometric measures were examined using Pearson's or Spearman's correlation coefficients, depending on data distribution. Correlation strength was classified according to Cohen (1992) [28], adopting the following benchmarks: *r* = 0.10 as small, *r* = 0.30 as medium, and *r* = 0.50 as large.

Correlations between the SyNCA total score and the following indicators were tested: AMC, HGS, total AMMI, upper limb AMMI (AMMI‐UL), and lower limb AMMI (AMMI‐LL). Construct validity was established if a correlation coefficient between 0.4 and 0.8 was observed between SyNCA scores and at least one of the external measures, in line with standards for health scale validation [29]. Correlations were assessed in the overall sample and stratified by sex. All analyses were two‐tailed, and a significance level of *p* < 0.05 was adopted. Statistical analyses were performed using the Statistical Package for the Social Sciences (SPSS®) version 21.

## 3 Results

### 3.1 Phase 1: SyNCA Development, Content and Semantic Validation (Delphi Protocol)

The first SyNCA version underwent two consecutive validation stages, focused on three core attributes: relevance, pertinence, and representativeness. Eighteen state‐registered clinical nutrition experts, each with over 6 years of post‐qualification experience and working in public hospitals across four regions of Brazil at the time of the validation stage, were invited to participate. In Stage 2 of content validation, 17 experts completed the questionnaire.

### 3.2 Validation Stage 1: Content Validation

Two evaluation cycles were required for full validation of the SyNCA content. In the first evaluation cycle, 4 of the 5 anatomical regions and 12 of the 18 proposed body segments achieved a CVI > 0.80 across all three attributes and were approved.

In the facial region, the ‘orbital region' item was excluded due to insufficient pertinence. In the lower limbs, the item ‘oedema around the malleolar region' was rejected on all three attributes. In the dorsal trunk, the ‘subscapularis & latissimus dorsi' and ‘paravertebral muscles' items were not approved for two attributes. In the lower limbs, the ‘quadriceps muscle' and ‘general inspection of lower limb bone structure' failed to meet the relevance threshold.

Based on these findings, removal of the dorsal trunk region and its unapproved items was initially suggested. However, the deltoid muscle received a satisfactory CVI, and the region was therefore retained for reassessment in the next cycle.

The second evaluation cycle re‐evaluated the reconfigured instrument layout, including the team's proposal to maintain the dorsal trunk region and the two lower limb items previously not approved. Attributes with unsatisfactory CVI values were reviewed and revised based on expert feedback and team decisions. Changes in opinions for some experts were noted between the first and second evaluation cycles when assessing the five anatomical regions and the 18 body segments that were initially included in the SyNCA instrument.

Following the second evaluation cycle, the items ‘malleolar region', ‘subscapularis & latissimus dorsi', and ‘paravertebral muscles' were removed due to CVI values below the required threshold for relevance and representativeness. Combined with the earlier exclusion of the ‘orbital region', a total of four items were eliminated due to their relevance and representativeness. The final SyNCA version after content validation included five anatomical regions and 14 body segment items.

### 3.3 Validation Stage 2: Validation of Multiple‐Choice Answers and Semantic Analysis

Stage 2 involved a single evaluation cycle, where the invited experts assessed the multiple‐choice answer options for each body segment in terms of relevance, pertinence, and clarity. The dorsal trunk region was retained solely with the deltoid muscle, which achieved a CVI of 0.82 across all attributes. All remaining 14 items and their respective multiple‐choice answer options achieved CVIs ranging from 0.82 to 1.00 and were approved.

Semantic analysis was conducted with 14 registered nutritionists in their second year of the Clinical Nutrition Residency Program at the School of Nutrition, Bahia Federal University Teaching Hospital. All state‐registered nutritionists participating in this stage of the research were female, 70% of them had completed their undergraduate degree in Human Nutrition and Dietetics within one to 2 years, and 30% between three and 4 years.

The comprehension attribute was assessed for individual items (scored 0, 2, 4, or 6 points) and for the overall NDI total score. All items and the final score achieved full consensus. CVIs for item comprehension ranged from 0.90 to 1.0, and fK values ranged from 0.80 to 1.0, indicating strong agreement. With content and semantic validation complete, the final SyNCA version consisted of five anatomical regions and 14 body segment items, advancing to the next phase of construct validation.

### 3.4 Phase 2: Multicentre Validation of the SyNCA Instrument

A total of 136 hospitalised DCLD patients were included across three public hospitals in Brazil. Most patients were male (77.9%, *N* = 106), with average age 56.9 ± 11.5 years. The most frequent aetiology was alcoholic (47.8%, *N* = 65), and most patients had Child‐Pugh B classification (59.6%, *N* = 81). Ascites was detected in 68.9% (*N* = 93) of the sample. Out of those, 54.8% (*N* = 51) presented with moderate ascites. The prevalence of malnutrition was 75% (*N* = 102) according to AMC adequacy. Regarding HGS, 36.8% (*N* = 50) of patients had a score below the reference threshold for their sex. The clinical and nutritional characteristics of the DCLD sample used in construct validation are detailed in Table 2.

**Table 2:** Clinical and Nutritional Characterisation of DCLD Hospitalised Patients Assessed for SyNCA Construct Validation.

Variable	Total
	*N* = 136
Aetiology^a^	
Alcoholic	65.0 (47.8)
Undetermined	39.0 (28.7)
Viral	14.0 (10.3)
MASLD	13.0 (9.6)
Autoimmune	4.0 (2.9)
Primary Biliary Cirrhosis	1.0 (0.7)
Severity	
Child Pugh^a^	
A	26.0 (19.1)
B	81.0 (59.6)
C	29.0 (21.3)
MELD Na^b^	17.0 (12.0–21.0)
Ascites	
present	93.0 (68.9)
Ascites severity	
Mild	22.0 (23.7)
Moderate	51.0 (54.8)
Severe	20.0 (21.5)
Dynamometry	
HGS (kg)^b^	29.5 (20.3–38.2)
Nutritional status	
BMI (kg/m²)	22.3 (4.8)^c^
SyNCA	33.2 (18.8)^c^
AMC (cm)	21.7 (3.6)^c^
AMMI Total (kg/m²)	—

Synonyms: AMC, Arm Muscle Circumference; AMMI, Appendicular Lean Mass Index (kg/m²) (obtained by Dual‐energy X‐ray absorptiometry); BMI, Body Mass Index; HGS, Handgrip Strength; MASLD, Metabolic Dysfunction‐Associated Steatosis Liver Disease; MELD Na, Model for End‐stage Liver Disease Sodium; SyNCA, Systematised Nutritional Clinical Assessment.

^a^ Results presented as frequency and percentage.

^b^ Results presented as median and interquartile range (Q25–Q75).

^c^ Results presented as mean and standard deviation.

**Figure 2:**
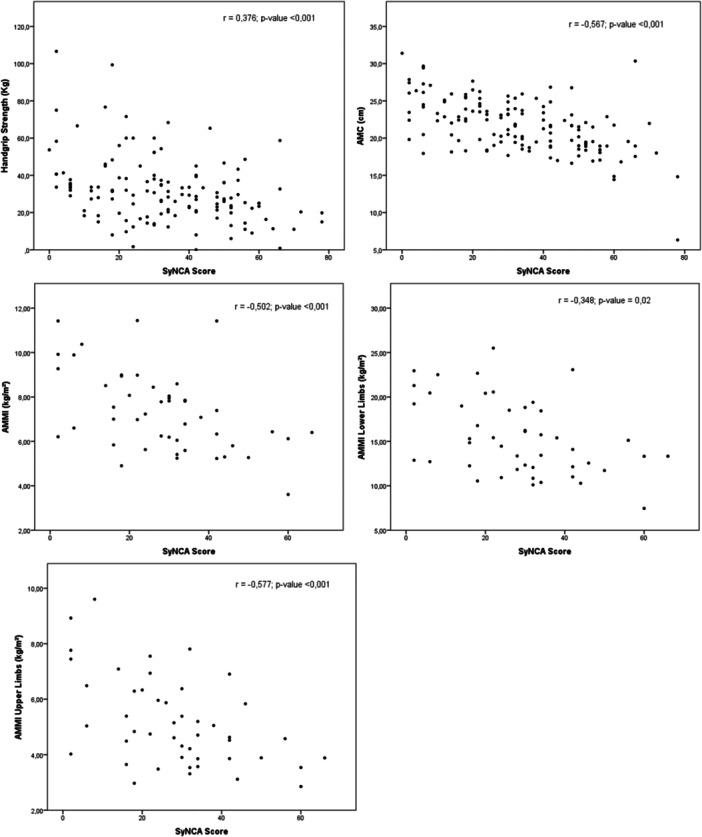
Correlation between SyNCA, BMI, AMC, HGS and AMMI in DCLD hospitalised patients. Legend: ^a^Pearson correlation coefficient; ^b^Spearman correlation coefficient; ^c^SyNCA scores for deltoid, biceps and triceps, adductor pollicis and dorsum of the hands; ^d^SyNCA scores for quadriceps, gastrocnemius, bone structures and oedema; Abbreviations: AMC, Arm Muscle Circumference; AMMI, Appendicular Muscle Mass Index (kg/m²) obtained by Dual‐energy X‐ray absorptiometry (DXA); LL, Lower Limbs; UL, Upper Limbs.

SyNCA achieved construct validity based on its correlation analysis with AMC (*r* = −0.567; *p* < 0.001), total AMMI (*r* = −0.502; *p* < 0.001), and AMMI‐UL (*r* = −0.577, *p* < 0.001). The instrument demonstrated statistically significant correlations with all tested variables. Moderate correlations were observed for AMC, HGS, and all AMMI parameters (Figure 2, Table 3).

**Table 3:** Correlation Outcomes Between SyNCA, BMI, AMC, HGS and AMMI in DCLD Hospitalised Patients.

Variables	Total			AMMI Subgroup		
	*N* = 136			*N* = 47		
SyNCA	BMI^a^	AMC^a^	HGS^b^	AMMI Total^a^	IMMA‐UL	IMMA‐LL
	−0.632*	−0.567*	−0.376*	−0.502**	—	—
SyNCA – UL^c^	—	—	—	—	−0.577**	—
SyNCA – LL^d^	—	—	—	—	—	−0.348***

Abbreviations: AMC, Arm Muscle Circumference; AMMI, Appendicular Muscle Mass Index (kg/m²) obtained by Dual‐energy X‐ray absorptiometry (DXA); BMI, Body Mass Index; HGS, Handgrip Strength; LL, Lower Limbs; SyNCA, Systematic Nutritional Clinical Assessment; UL, Upper Limbs.

* *p*‐value ≤ 0.0001;

** *p*‐value ≤ 0.001;

*** *p*‐value = 0.02.

^a^ Pearson correlation coefficient.

^b^ Spearman correlation coefficient.

^c^ SyNCA scores for deltoid, biceps and triceps, adductor pollicis and dorsum of hands.

^d^ SyNCA scores for quadriceps, gastrocnemius, bone structures and oedema.

Sex‐stratified analyses were conducted for AMC, HGS, and AMMI, with correlation coefficients between SyNCA and these variables presented separately for men and women. Additionally, a statistically significant positive correlation was found between AMC and total AMMI obtained by DEXA analysis, supporting the construct validity of SyNCA's musculoskeletal assessments (Supplementary Tables 2 and 3).

## 4 Discussion

This study outlines the development and validation of the Systematic Nutritional Clinical Assessment (SyNCA). Designed through a systematised protocol, SyNCA demonstrated validity across all assessment stages for content, semantic, and construct, proving its applicability and reliability in clinical settings for the nutrition‐focused physical examination of DCLD hospitalised patients.

The importance of using physical examination‐based tools such as the NFPE has been recognised in clinical practice [9, 30] and is supported by ASPEN guidelines for accurate nutritional assessment [10]. SyNCA builds on this foundation by offering a structured, validated instrument that enhances reproducibility and ease of use, especially in clinical settings where time, resources, or access to imaging technologies are limited.

The SyNCA validation process has adhered to methodologies aligned with international standards, including the use of the CVI as recommended in the Consensus‐based Standards for the selection of health Measurement Instruments (COSMIN) checklist for health measurement instruments [27]. Our approach has mirrored the development of other validated instruments [31, 32], incorporating expert consensus for item inclusion or exclusion [33]. Most SyNCA items achieved CVI scores of ≥ 0.80, supported by strong agreement via the obtained fK coefficient, indicative of consensus among the clinical nutrition experts invited to contribute to our study.

Some NFPE components, including the orbital, subscapular, latissimus dorsi, and paravertebral regions, were excluded from SyNCA. While these are traditionally part of the NFPE, their removal simplified our instrument without compromising its validity. Our experience has shown that this streamlining has improved SyNCA's viability in bedside practice, particularly for critically ill or bedridden patients, where access to the subscapular, latissimus dorsi, and paravertebral regions was limited or just not possible.

Following Delphi recommendations, semantic analysis was conducted with frontline professionals to test the clarity and clinical applicability of the tool, as previously recommended [34]. The state‐registered nutritionists invited to contribute to this study demonstrated strong consensus on the comprehension and usability of the instrument, reflected in high CVI and fK scores for all 14 SyNCA items.

Previous studies have highlighted subjectivity as a limitation in NFPE‐based assessments [35], which SyNCA has addressed through its structured design. The correlations found between SyNCA scores and established markers of nutritional status, such as AMC, were strong and statistically confirmed. Our findings are consistent with previous research showing AMC as a reliable marker of malnutrition in patients with liver cirrhosis [36]. Additionally, SyNCA scores were inversely correlated with DEXA‐determined arm and leg AMMI, reinforcing the instrument's capacity to identify muscle depletion.

Ascites complicates nutritional assessment in DCLD, as fluid overload distorts body composition metrics. Our study mitigated the issue of fluid overload by excluding the trunk region in DEXA analyses, avoiding lean mass overestimation. This approach aligns with recommendations suggesting the use of limb‐based measurements in ascitic patients [37]. Our results further confirm that limb‐specific AMMI is a reliable correlate of SyNCA scores, strengthening its validity in DCLD populations.

Notably, previous work has shown that DEXA‐determined arm muscle mass is a better predictor of sarcopenia‐related mortality than total appendicular muscle mass [38, 39]. Our findings agree with previous research, as SyNCA scores correlated well with upper limb muscle mass, supporting its diagnostic accuracy.

Physical examination remains particularly relevant for the identification of malnutrition in patients presenting with fluid retention, such as oedema and ascites, common occurrences in liver and kidney disease [40, 41]. Given its strong correlation with multiple objective markers, SyNCA represents a reliable bedside tool for nutritional diagnosis using standardised methods.

SyNCA's performance mirrors findings of other studies comparing subjective and objective nutritional assessment tools. For instance, Gbareen, Barnoy, and Theilla (2021) [42] observed in a clinical study conducted with 228 patients with chronic diseases a significant agreement between a subjective (Subjective Nutritional Assessment, SNA) and an objective (Mini‐Nutritional Assessment, MNA) tool, reinforcing the utility of structured clinical assessment tools in the absence of advanced diagnostic instruments.

It is noteworthy that studies which have evaluated the correlation between subjective tools and objective methods for nutritional status assessment demonstrate a strong correlation between the instruments [43, 44, 45]. However, many of the evaluative methods used in these comparisons are themselves components of the instruments being assessed, which may influence the high correlation values observed. Our instrument is entirely novel and original and is not derived from any pre‐existing tool used in clinical practice. This may account for the moderate, rather than strong, correlations found.

The lower proportion of women comprising our sample may have influenced the results of the analyses performed for this group. This phenomenon has been noted in other studies involving individuals with chronic liver disease, where a lower proportion of women affected the significance of comparisons between two objective methods (SMI by BIA or computed tomography) [46]. For example, a clinical study involving 268 patients diagnosed with cirrhosis, of whom 67% were male, demonstrated significant differences in body composition between males and females. The findings indicated significant protein depletion in 51% of the patients, which was more prevalent in men than in women, irrespective of liver disease severity or aetiology [47]. A clinical study conducted with 795 patients with hepatic cirrhosis, of whom 70.6% were male, utilised computed tomography to measure muscle indices (PSMI, AWMI, SMI) and identify predictors of disease complications. The results demonstrated that loss of paravertebral muscle mass (PSMI) and of the skeletal muscle index (SMI) was associated with disease severity and complications exclusively in male patients, but not in females [48]. A recent study of 382 patients equally distributed between males and females showed that the prevalence of SMI‐defined sarcopenia was significantly higher in men than in women, with the authors reporting a positive correlation between reduced SMI and lower quality of life exclusively in the male patients [49]. The findings summarised above corroborate our findings and collectively suggest that specific gender‐related factors may influence the nutritional status of patients with cirrhosis in a divergent manner, reinforcing the necessity of a patient‐centred approach in the assessment of nutritional status and body composition abnormalities.

Early and accurate nutritional diagnosis can lead to timely interventions, reducing complications and improving patient outcomes [40]. One of the key strengths of our study is the innovative development of a reliable, low‐cost, and reproducible clinical instrument. SyNCA's low‐cost and rapid application features are especially relevant in underfunded public hospitals, where advanced imaging is rarely available [50, 51].

SyNCA development has followed the methodological guidance of the COSMIN initiative [27] and will be subjected to further validation studies, including criterion validity and responsiveness for other chronic conditions. This phased validation approach has been used in similar frameworks, such as the GLIM criteria, which published content validation prior to full implementation [52, 53].

In conclusion, SyNCA offers a practical, validated solution for nutritional diagnosis, particularly in clinical contexts where imaging, anthropometry, or bioimpedance may not be viable due to fluid overload or patient immobility. Its relevance is heightened in public healthcare settings, where underfunded and understaffed hospital systems with limited access to complex diagnostic tools are common. SyNCA supports the delivery of early and reliable nutritional diagnosis through systematised nutrition‐focused physical examination, bridging the gap between subjective and objective assessment in frontline clinical practice.

## Author Contributions

Rosangela Passos de Jesus, Lucivalda Pereira Magalhães de Oliveira, Andre de Castro Lyra, Fernando Gomes Romero, and Allain Amador Bueno contributed to conceptualization and Methodology.All authors contributed to investigation, data curation, and formal analysis.All authors contributed to writing – original draft and writing – review & editing.All authors approved the final version of the manuscript and agree to be accountable for all aspects of the work, ensuring its integrity and accuracy.

## Ethics Statement

The study received approval from the Research Ethics Committees of the three participating hospitals in Brazil, namely Professor Edgard Santos University Hospital Complex (HUPES, Bahia Federal University, Salvador, Brazil); Bahia State Roberto Santos General Hospital (HGRS, Salvador, Brazil); and Clinical Hospital of the Medical Faculty of Botucatu (HCFMB, Sap Paulo, Brazil). All participants in both phases of the study voluntarily agreed to take part and signed a standardised Free and Informed Consent Form.

## Consent for Publication

All authors give their consent for this study to be published.

## Conflicts of Interest

The authors declare no conflicts of interest.

## Supporting information


**Supplementary Table 1:** Systematic Nutritional Clinical Assessment (SyNCA) Instrument.

**Supplementary Table 2:** Correlation between SyNCA and Arm Muscle Circumference, Handgrip Strength, and Appendicular Muscle Mass Index in hospitalised patients with Chronic Liver Disease, stratified by sex.
**Supplementary Table 3:** Correlation between SyNCA and the Appendicular Muscle Mass Index of the upper and lower limbs in hospitalised patients with Chronic Liver Disease, stratified by sex.
