# Supplementary material for: Systematic Nutritional Clinical Assessment (SyNCA): Instrument Development, Delphi Protocol for Content, and Semantic Validation
Source: J Hum Nutr Diet. 2026 Mar 24;39(2):e70235. doi: 10.1111/jhn.70235 (PMC13013094; doi:10.1111/jhn.70235)
Supplement: Supplementary file 1 — Supplementary Table 1: Systematic Nutritional Clinical Assessment (SyNCA) Instrument. [file JHN-39-0-s002.docx]

Supplementary Table 1: Systematic Nutritional Clinical Assessment (SyNCA) Instrument

| **Body Region Assessed** | | **Score for body region assessed** | | | |
| --- | --- | --- | --- | --- | --- |
|  |  | **Score = 0** | **Score = 2** | **Score = 4** | **Score = 6** |
| **Muscle and other anatomical structures** | | **Justification:** Presence of well-preserved muscular and adipose tissue, with no prominence of bony structures.  Body structures adequately covered with muscle, adipose tissue and skin of normal thickness. | **Justification:** Slight muscular and adipose tissue depletion.  Slightly apparent bony structures, but not prominent, covered with muscle, adipose tissue and skin of normal thickness. | **Justification:** Moderate depletion of muscular and adipose tissue, with moderate view of bony structures.  Moderately apparent bony structures, slightly prominent, mostly covered with adipose tissue and thinning skin. | **Justification:** Marked depletion of muscle and adipose tissue, with marked prominence of bony structures.  Markedly apparent and prominent bony structures, mostly covered by thin skin. |
| **Face** | **Temporalis – Muscle of the temples**  **Scoring:**  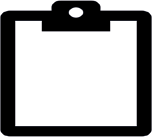 | Straight and flat region without clear indication of temple concavity.  The well-defined muscle can be felt on palpation. | Region with slightly apparent concavity of the temples.  It is possible to feel the slight tissue depression near the temples, but the muscle is noticeable. | Region with moderately apparent concavity of the temples.  It is possible to feel moderate tissue thinning near the temples, however the muscle is barely perceptible. | Region with markedly apparent concavity of the temples.  Marked tissue depletion with prominent bony structures, muscle is not noticeable. |
|  | **Masseter – Chewing muscle**  **Scoring:**  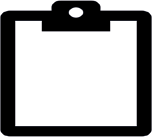 | Facial region with rounded contour.  Muscle easily perceptible through palpation, regardless of mastication simulation. | Facial region with elongated contour and bony structures with slight prominence.  Mild depletion of muscle structures can be noticed when the patient simulates mastication. | Facial region with angular contour and bony structures with moderate prominence.  Moderate depletion in muscle thickness can be noticed when the patient simulates mastication. | Facial region with more angular contour and bony structures with accentuated prominence.  A marked depletion in muscle thickness can be noticed when the patient simulates mastication. Muscle movement is nearly imperceptible. |
|  | **Bichat fat pad – Cheek fat pad**  **Scoring:**  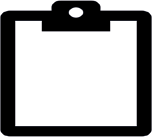 | Bichat fat pad can be visualized independently of smile simulation.  Accentuated protrusion of the cheeks with smile simulation.  See figure 01 for demarcation of specific areas for cheek fat pad assessment | Bichat fat pad can be visualized only with smile simulation.  Moderate protrusion of cheeks with simulated smile. | Moderate reduction of the Bichat fat pad seen during smile simulation.  Slight protrusion of the cheeks with smile simulation. | Marked reduction of Bichat fat pad independent of smile simulation.  Presence of concavity on the side of the face between the mandible and the zygomatic bone.  Absence of cheek protrusion with smile simulation. |
|  | **Zygomatic arch and mandible lines**  **Scoring:**  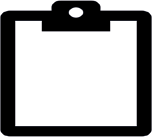 | The bony angulations formed by the orbital rim, zygomatic bone and mandible lines are not noticeable. Infra-orbital compartment of the eyes features adequate presence of adipose tissue.  Unable to observe sharp angulation along the orbital rim.  See figure 02 | Presence of slightly perceptible angulations in the zygomatic bone and mandible.  Unable to observe sharp angulation along the orbital rim. | Presence of moderately perceptible angulations in the zygomatic bone, maxilla and mandible.  The sharp angulation along the orbital rim is not fully noticeable. | Presence of strikingly perceptible angulations in all facial bones.  The sharp angulation along the orbital rim is easily perceptible bilaterally, with prominence of bony structures, depletion of facial muscles and reduction of subcutaneous tissue. |
| **Body Region Assessed** | | **Score = 0** | **Score = 2** | **Score = 4** | **Score = 6** |

| **Muscle and other anatomical structures** | | **Justification:** Presence of well-preserved muscular and adipose tissue, with no prominence of bony structures.  Body structures adequately covered with muscle, adipose tissue and skin of normal thickness. | **Justification:** Slight muscular and adipose tissue depletion.  Slightly apparent bony structures, but not prominent, covered with muscle, adipose tissue and skin of normal thickness. | **Justification:** Moderate depletion of muscular and adipose tissue, with moderate view of bony structures.  Moderately apparent bony structures, slightly prominent, mostly covered with adipose tissue and thinning skin. | **Justification:** Marked depletion of muscle and adipose tissue, with marked prominence of bony structures.  Markedly apparent and prominent bony structures, mostly covered by thin skin. |
| --- | --- | --- | --- | --- | --- |
| **Thorax: Frontal Region** | **Trapezius and Pectoralis Major – Muscles covering the shoulder, clavicle and chest**  **Scoring:**  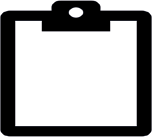 | Rounded shoulder contour with adequate reserve of muscle and subcutaneous tissue.  Men: bony structures are not apparent.  Women: constitutively, such bony structures are noticeable but not prominent.  See figure 03 | Rounded shoulder contour, however, there is slight depletion in muscle thickness.  Men: slightly apparent bony structures.  Women: slightly prominent bony structures. | Angular shoulder contour due to moderate depletion in muscle thickness.  Men: visible bony structures.  Women: Moderately prominent bony structures. | Outline of shoulders with prominent angulation due to marked depletion in muscle thickness.  Very prominent bony structures in both sexes. |
|  | **Intercostal muscles – muscles covering ribs and around sternum**  **Scoring:**  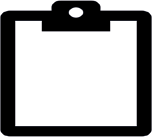 | Muscles have a preserved shape and can be easily noticed.  The bony structures are completely covered by muscle, adipose tissue and skin of normal thickness.  Upon deep inspiration, muscle movement is clearly perceptible. In patients with ascites, observe figure.  See figure 04 | Muscles are slightly reduced in thickness and elasticity but can still be easily noticed.  The bony structures are covered by muscles, adipose tissue and skin of normal thickness.  Upon deep inspiration, muscle movement with slightly visible ribs is observed. | Muscles are moderately reduced in thickness and elasticity but can still be noticed.  Bony structures are mainly covered by adipose tissue and thinning skin.  Upon deep inspiration, muscle movement is less obvious, with moderately apparent ribs. | Muscles are markedly reduced in thickness and elasticity, barely perceptible on palpation.  The bony structures are mainly covered by thin skin.  Upon deep inspiration, imperceptible muscle movement and markedly apparent ribs are observed. |
| **Thorax: Dorsal Region** | **Deltoid – Muscle covering acromion, acromial angle of the scapula and upper part of humerus**  **Scoring:**  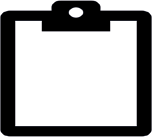 | The muscle has a preserved, inverted delta (Δ) shape and can be easily visualized, even without lateral movement of the arm.  Rounded shoulders due to proper shape and thickness of the deltoid muscle adequately covering the acromion.  See figures 05 e 06 | The muscle has preserved shape, muscle contraction during lateral movement of the arm is easily perceptible.  Less rounded shoulders, due to slight reduction in deltoid thickness. | The muscle presents a delta-shaped change, and muscle contraction during lateral movement of the arm is barely perceptible.  Angular shoulders, due to moderate reduction in deltoid thickness. | The muscle presents a marked change in its delta shape, and muscle contraction during arm movement is almost imperceptible.  Square shoulders due to well-defined angles and prominent acromion.  A marked reduction in deltoid thickness is observed. |
| **Upper Limbs** | **Biceps and Triceps – Arm muscles**  **Scoring:**  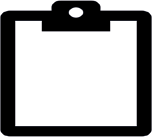 | The muscles have preserved format and can be easily perceived by visual inspection, palpation and techniques performed.  See figure 07 | The muscles are slightly depleted but can be perceptible during palpation and techniques performed.  Inspection reveals mild depletion in muscle thickness. | The muscles are moderately depleted, with impairment of perception during palpation and techniques performed.  Inspection reveals moderate depletion in muscle thickness and bony structures are perceptible | The muscles show accentuated depletion and expressive impairment of perception during palpation and techniques performed.  Inspection reveals marked depletion in muscle thickness and bony structures are prominent. |
|  | **Adductor pollicis muscle – the thumb muscle**  **Scoring:**  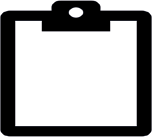 | The muscle has preserved shape and thickness and can be easily visualized, even without movement.  Presence of protrusion, easily identifying the muscle.  See figure 08 | The muscle has preserved shape and thickness and can be visualized even without movement.  Reduction of muscle protrusion, indicating mild depletion in muscle thickness. | The muscle presents depleted shape and thickness, with impaired perception during movement.  A concavity can be noticed in the space between the index and thumb.  Inspection demonstrates bony structures of the index finger and thumb. | The muscle presents severely depleted shape and thickness. Significant impairment of perception during movement.  A marked concavity can be noticed in the space between the index finger and the thumb.  Inspection demonstrates prominent bony structures of the index finger and thumb. |

| **Body Region Assessed** | | **Score = 0** | **Score = 2** | **Score = 4** | **Score = 6** |
| --- | --- | --- | --- | --- | --- |
| **Muscle and other anatomical structures** | | **Justification:** Presence of well-preserved muscular and adipose tissue, with no prominence of bony structures.  Body structures adequately covered with muscle, adipose tissue and skin of normal thickness. | **Justification:** Slight muscular and adipose tissue depletion.  Slightly apparent bony structures, but not prominent, covered with muscle, adipose tissue and skin of normal thickness. | **Justification:** Moderate depletion of muscular and adipose tissue, with moderate view of bony structures.  Moderately apparent bony structures, slightly prominent, mostly covered with adipose tissue and thinning skin. | **Justification:** Marked depletion of muscle and adipose tissue, with marked prominence of bony structures.  Markedly apparent and prominent bony structures, mostly covered by thin skin. |
| **Upper Limbs** | **Back of hands, interosseous muscles**  **Scoring:**  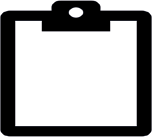 | Back of hand adequately covered by evidently noticeable muscles and subcutaneous tissue. Carpus, metacarpus and phalanges are not easily perceived.  Firm and vigorous handshake. | Back of hand covered by fairly noticeable muscles and subcutaneous tissue. Carpus, metacarpus and phalanges can be perceived.  Firm handshake. | Back of hand shows reduced subcutaneous cellular tissue. Bony structures of the carpus, metacarpus and phalanges can be perceived. Inspection shows prominence of hand bones.  Handshake with moderate intensity. | Back of hand shows significantly reduced subcutaneous cellular tissue. Accentuated perception of the bony structures of the carpus, metacarpus and phalanges. Inspection shows very prominent bony structures of the hands.  Handshake with light intensity. |
| **Lower Limbs** | **Quadriceps – Muscles located on the front of the thigh**  **Scoring:**  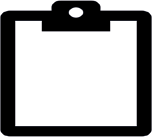 | Muscle with adequate thickness, firm muscle fascia and adequately covered bony structures.  Well-preserved muscle, completely covering the femur. It can be visualized easily, without the need for thigh palpation during leg extension.  See figure 09 | Muscle with slight reduction in thickness, firm muscle fascia and covered bony structures.  Well-preserved muscle lining the femur. It can be easily identified by palpating the thigh during leg extension. | Muscle with moderate thickness depletion, flaccid muscle tissue and apparent bony structures.  Smaller muscle volume in the inner and anterior part of the thigh, making the femur more apparent.  Muscle contraction can still be observed with deep thigh palpation during leg extension. | Muscle with marked depletion in thickness and no discernible muscle tissue. The thigh region has excess skin.  Muscle with reduced dimension throughout the thigh, leaving the epiphyses of the femur and the patella clearly prominent.  Muscle movement imperceptible even on deep thigh palpation during leg extension. |
|  | **Gastrocnemius – Muscle that covers the back of the leg, below the knee**  **Scoring:**  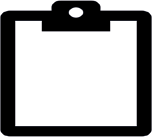 | The muscle has an adequate shape, calf prominence and well-preserved and noticeable muscle tissue. Anatomical divisions such as the lateral and medial heads of the gastrocnemius can be easily identified on palpation.  Muscle contraction is perceptible on visual inspection without the need for palpation during foot flexion.  See figure 10 | The muscle shows slight depletion in thickness, slight reduction in calf prominence and less firm muscle tissue. The lateral and medial heads of the gastrocnemius can be identified on palpation but it shows slight flaccidity.  Muscle contraction can be easily perceptible on palpation during foot flexion. | The muscle shows moderate thickness depletion, less calf prominence, with less noticeable muscle tissue. Upon palpation, moderate muscle flaccidity is observed, with difficulty identifying the lateral and medial heads of the gastrocnemius.  Muscle contraction can still be perceptible on palpation during foot flexion, but the region is less firm. | The muscle shows marked depletion in thickness, expressive reduction of calf prominence and non-perceptible muscle tissue. Upon palpation, marked muscle flaccidity is observed without definition of anatomical divisions.  The bony structures of the legs are prominent and the region has excess skin.  Muscle contraction is imperceptible to palpation during foot flexion and the region is not firm. |
|  | **General inspection of the bony structures of the lower limbs**  **Scoring:**  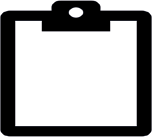 | Bony structures of lower limbs and iliac crest not apparent or prominent. Hip bones, legs, knees, ankles and feet adequately covered by muscle, subcutaneous tissue and skin.  Malleolar region without signs of inflammation and edema related to nutritional status, and adequately covered by muscles, subcutaneous tissue and skin. | Bony structures of lower limbs and iliac crest slightly apparent, but not prominent and without visible bony angulations. Bones covered by muscles, subcutaneous cellular tissue and skin.  Malleolar region without signs of inflammation and edema related to nutritional status, and covered by muscles, subcutaneous tissue, and skin. | Bony structures of lower limbs and iliac crest moderately apparent and prominent, allowing visualization of bony angulations. Bones covered by a thin layer of subcutaneous tissue and presence of excess skin, usually with flaccidity.  Malleolar region without signs of inflammation and edema related to nutritional status but may show tissue depletion around the ankle with apparent bony structures. | Bony structures of the lower limbs and iliac crest are markedly apparent and prominent, allowing visualization of more pronounced bony angulations. Bones covered by very thin layer of subcutaneous tissue and excess skin with evident flaccidity.  The malleolar region may show signs of inflammation and edema related to the nutritional status, with tissue depletion around the ankle making the bony structures very apparent. |
|  | **Presence of oedema in the lower limbs associated with underlying disease**  **Scoring:**  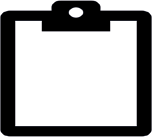 | Absence of edema associated with underlying disease.  Skin with maintained turgor and elasticity, without formation of depression at the site, no Godet sign after finger pressure.  See figure 11. | Mild edema associated with disease.  Bilateral interstitial fluid infiltration, located in the legs.  Skin with maintained turgor and elasticity, without signs of inflammation such as pain, heat, and redness.  Presence of superficial Godet sign: +/IV | Moderate edema associated with disease.  Bilateral interstitial fluid infiltration, located throughout the lower limbs.  Skin with maintained turgor and elasticity, which may show signs of inflammation.  Presence of deep Godet sign: ++ or +++/IV | Severe edema associated with disease.  Bilateral interstitial fluid infiltration, located along the entire length of the lower limbs up to the pelvic girdle.  Skin usually showing signs of inflammation and reduced turgor and elasticity.  Presence of very deep Godet sign: ++++/IV |

| **Total Score - Nutritional Depletion Index (NDI)**  **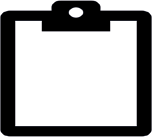** | **NDI = 0 to 21 points**    **(Percentile ≤ 25th)**  **No Nutritional Depletion** | **NDI = 22 to 42 points**    **(Percentile >25th - ≤ 50th)**  **Mild Nutritional Depletion** | **NDI = 43 to 63 points**    **(Percentile >50th - ≤ 75th)**  **Moderate Nutritional Depletion** | **NDI = 64 to 84 points**    **(Percentile > 75th)**  **Severe Nutritional Depletion** |
| --- | --- | --- | --- | --- |
| **Nutritional Diagnosis:** |  |  |  |  |
| **Legend:** Nutritional Depletion Index (NDI)  **Nutritional Diagnosis:**   1. Calculate the sum of the scores obtained from patient examination in each inspected body segment to obtain the NDI. 2. Identify the corresponding NDI to determine nutritional diagnosis.   To obtain nutritional diagnosis, the instrument’s total score has been segmented into quartiles:   - NDI between 0 and 21 points, percentile ≤25th, “no nutritional depletion”; - NDI between 22 and 42 points, between percentiles >25th to ≤50th, “Mild Nutritional Depletion”; - NDI between 43 and 63 points, between the >50th to ≤75th percentile “Moderate Nutritional Depletion”; - NDI between 64 and 84 points, greater than the >75th percentile “Severe Nutritional Depletion”.   **Glossary:**  **Bony angulation** – Greater prominence of bony structures due to skin flaccidity, muscle depletion, volumetric reduction of fat compartments, with consequent change of the contour tending to the formation of angles due to a more squared shape.  **Inspection** – Visual examination of the body area to be evaluated for identification of clinical signs, such as the presence of edema, muscle atrophy, reduction of adipose tissue, bony angulations, etc.  **Palpation** – Involves the sense of touch to assess the strength, elasticity, roughness, texture, and mobility of subcutaneous cellular tissue.  **Subcutaneous cellular tissue (SCT)** – Formed by two layers, one of connective tissue and the other of fat, which vary in thickness in different regions of the body. When subcutaneous cellular tissue is subjected to wide volumetric variations resulting from the loss of body weight, macroscopic alterations are observed.  **Protrusion** – Normal or pathological displacement of a body structure, producing protuberance, transient or permanent, at a body site. | | | | |

Representation of body regions and techniques used to assess muscle and subcutaneous cellular tissue (SCT) reserve.

| 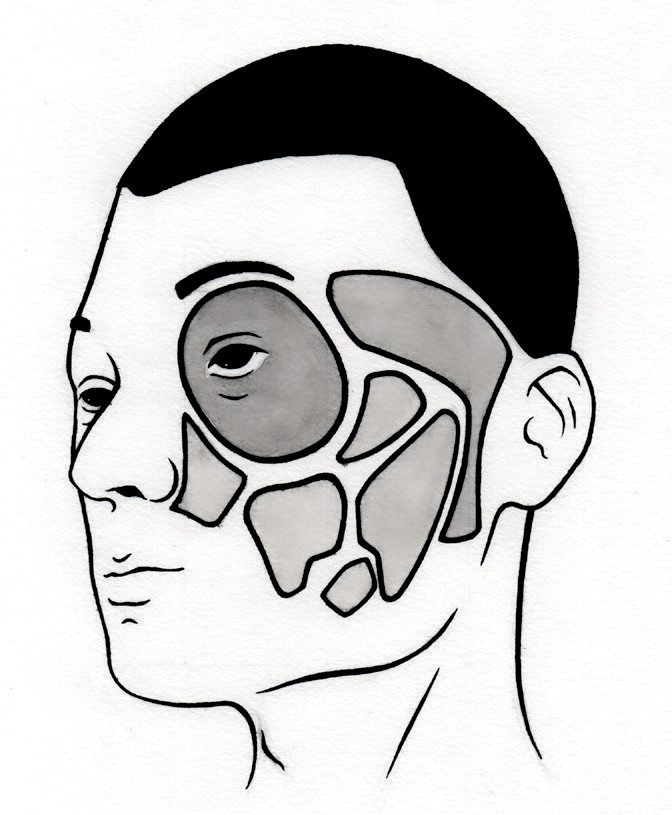  **Figure 1: Face with demarcation of specific areas for SCT assessment**  Well-preserved adipose tissue and bony structures adequately covered by muscle, adipose tissue and skin. | 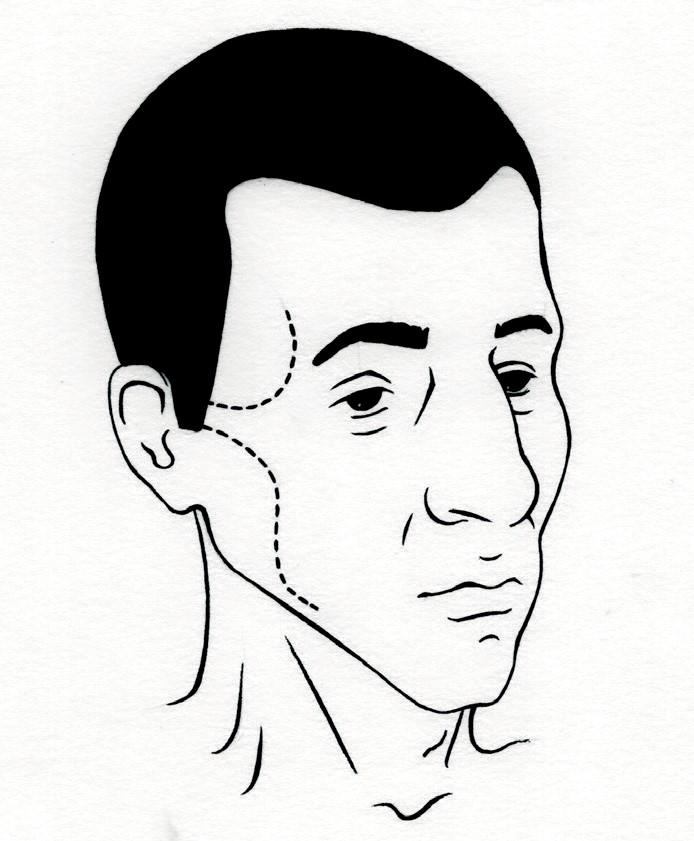  **Figure 2: Demarcation of areas assessed for identification of muscular atrophy with exposure of the zygomatic arch and mandible**  Prominence of bony structures due to sagging skin, muscle depletion, fat reduction and alteration of the bone contour with a rectangular shape. | 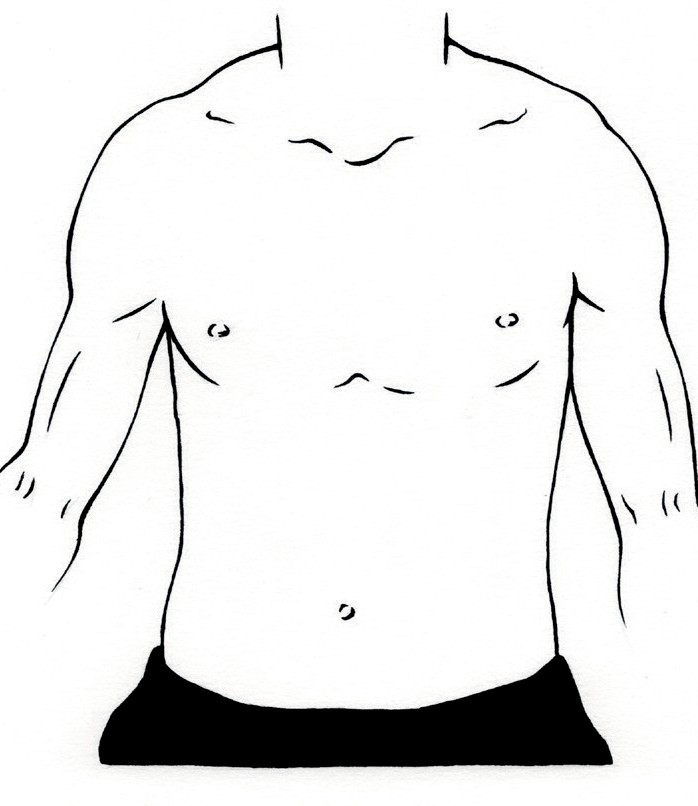  **Figure 3: Thorax, frontal region**  In this region, assess mainly the trapezius, pectoralis major and intercostal muscles. |
| --- | --- | --- |
| 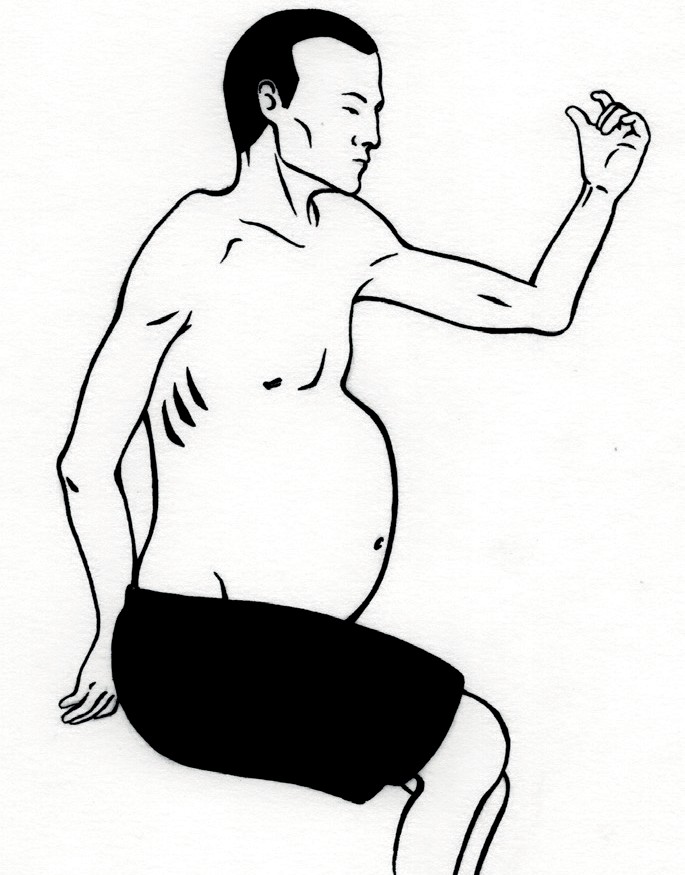  **Figure 4: Method to assess the intercostal muscles in patients with ascites**  To visualize these muscles, the patient must be positioned in lateral decubitus. Ask the patient to breath in deeply and hold for approximately 5 seconds. | 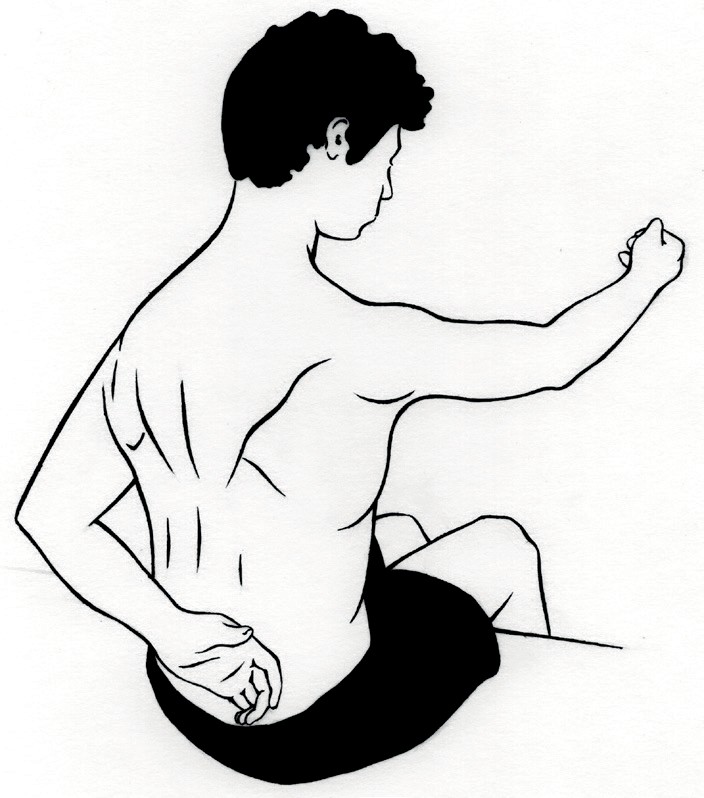  **Figure 5: Method for muscle inspection of the dorsal thorax**  The patient rotates the left forearm towards the lumbar region forming a 90-degree angle with the arm. Subsequently, the patient keeps the right arm in frontal extension, opening and closing their hands alternately. Observe the level of muscle movement. When this is not possible, the patient should be positioned in lateral decubitus with their back to the investigator. | 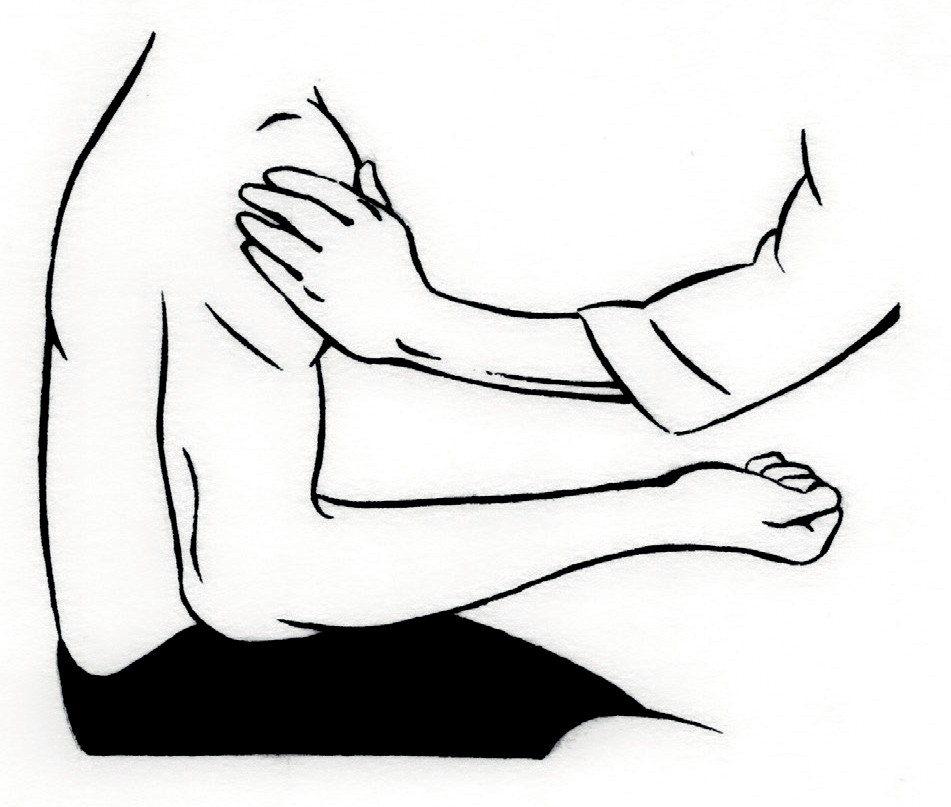  **Figure 6: Method for inspection of the deltoid**  The patient should preferably be sitting or standing with arms parallel along the body. Ask the patient to make repetitive arm extension and lateral flexion movements, while the examiner palpates the acromial region. |

| 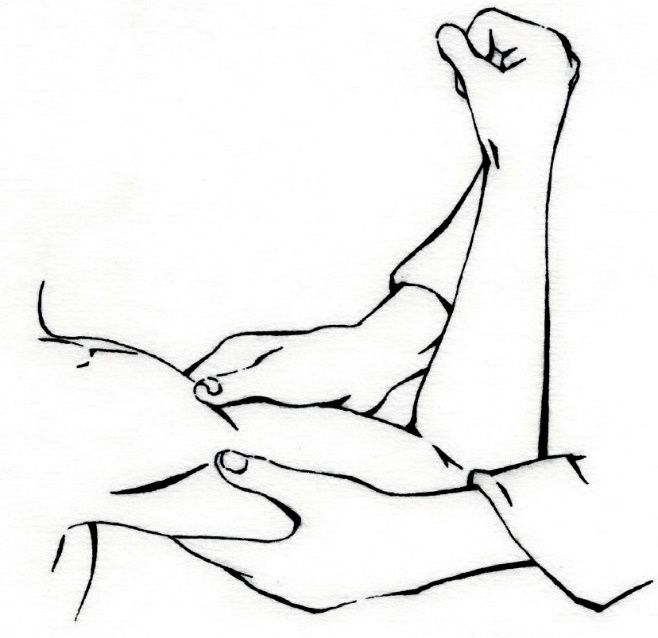  **Figure 7: Method for inspection of the biceps and triceps**  The patient should be sitting or standing with arms parallel along the body. Ask the patient to make a frontal flexion movement of the arm for hand inspection. | **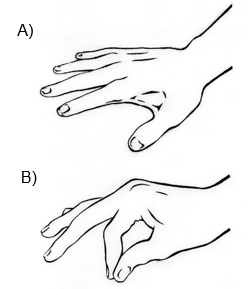**  **Figure 8: Method for inspection of the adductor pollicis**  Ask the patient to position the hand resting on a flat surface. Ask the patient to pinch with the thumb and index finger. Palpate the adductor pollicis muscle. | 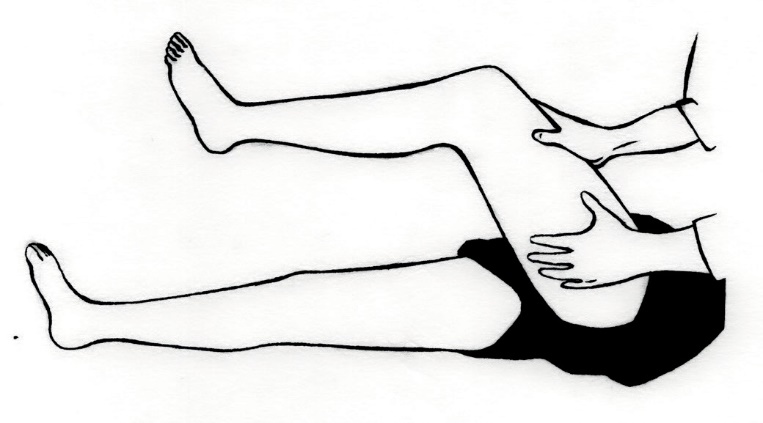  **Figure 09: Method for inspection of the quadriceps**  The patient should preferably be lying on the bed with one leg extended and the other flexed with the foot resting on the bed, forming an acute angle of approximately 40 degrees.  Ask the patient to make alternating leg extension and flexion movements, while the examiner palpates the thigh to assess muscle shape and thickness, and to identify muscle movement. |
| --- | --- | --- |
| 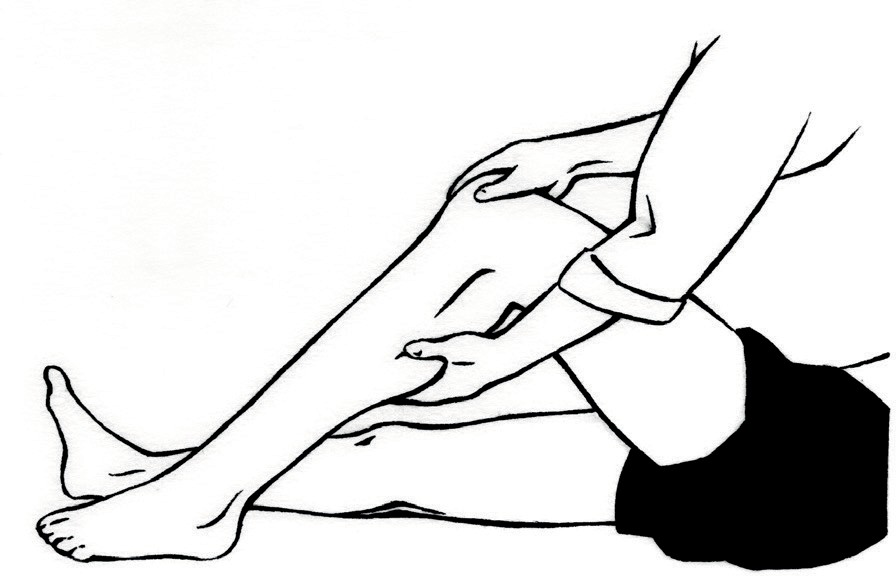  **Figure 10: Method for inspection of the gastrocnemius**  The patient must be lying on the bed with one leg extended and the one to be examined, with the knee flexed and the foot resting on the bed forming an acute angle of approximately 40 degrees. Perform the visual inspection in this initial, neutral position.  Subsequently, the patient should dorsiflex the foot while the examiner palpates the calf to assess muscle shape and thickness, and to identify muscle movement. | 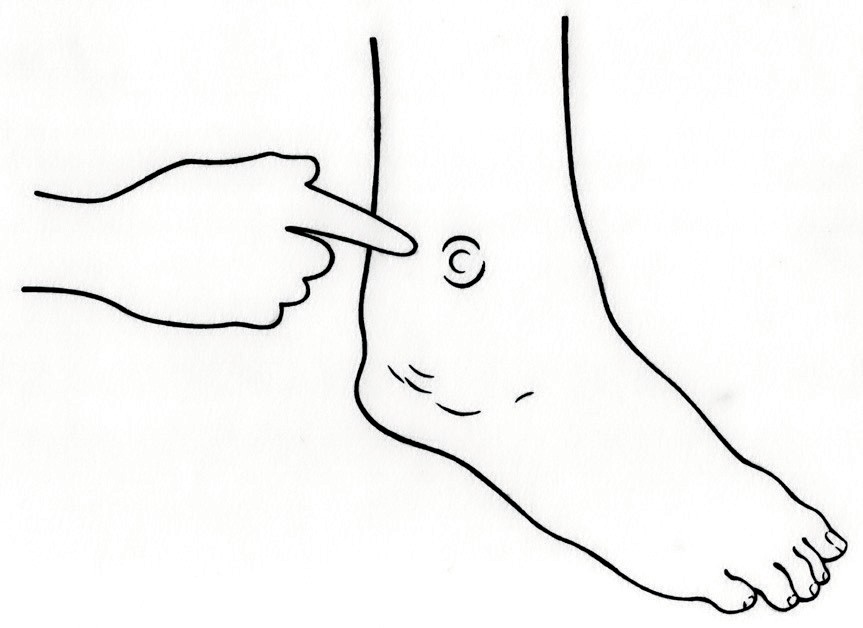  **Figure 11: Method for inspection of the malleolar region**  The patient must be lying in bed, with the foot resting on the bed. Evaluate the shape, muscle thickness and SCT.  Perform a firm, sustained compression of the site against the underlying structures, such as the tibia, fibula or medial malleolus, to identify the presence of edema. | **Comments:**  ____________________________________________________________________________________________________________________________________________________________________________________________________________________________________________________________________________________________________________________________________________________________________________________________________________________________________________________________________________________________________________________________________________________________________________________________________________________________________________________________________________________________________________________________________________________________________________________________________________________________________________________________________ |
