# Supplementary material for: Systematic Nutritional Clinical Assessment (SyNCA): Instrument Development, Delphi Protocol for Content, and Semantic Validation
Source: J Hum Nutr Diet. 2026 Mar 24;39(2):e70235. doi: 10.1111/jhn.70235 (PMC13013094; doi:10.1111/jhn.70235)
Supplement: Supplementary file 2 — Supplementary Table 2: Correlation between SyNCA and Arm Muscle Circumference, Handgrip Strength, and Appendicular Muscle Mass Index in hospitalised patients with Chronic Liver Disease, stratified by sex. Supplementary Table 3: Correlation between SyNCA and the Appendicular Muscle Mass Index of the upper and lower limbs in hospitalised patients with Chronic Liver Disease, stratified by sex. [file JHN-39-0-s001.docx]

**Supplementary Table 2:**Correlation between SyNCA and Arm Muscle Circumference, Handgrip Strength, and Appendicular Muscle Mass Index in hospitalised patients with Chronic Liver Disease, stratified by sex.

| Variables | SNS | | | |
| --- | --- | --- | --- | --- |
|  | Female  n=30 | p-value | Male  n=106 | p-value |
| AMC^a^ | -0.259 | **<0.001** | -0.654 | **<0.001** |
| HGS^b^ | -0.552 | **0.002** | -0.462 | **<0.001** |
| AMMI^a^* | -0.013 | 0.969 | -0.627 | **<0.001** |

Legend: ^a^ Pearson's correlation coefficient; ^b^ Spearman's correlation coefficient; * female n=12, male n=35. Abbreviations: AMC: Arm Muscle Circumference (cm); HGS: Handgrip Strength (kg); AMMI: Appendicular Muscle Mass Index (kg/m²) obtained by Dual-energy X-ray Absorptiometry; SyNCA: Systematic Nutritional Clinical Assessment.

**Supplementary Table 3:** Correlation between SyNCA and the Appendicular Muscle Mass Index of the upper and lower limbs in hospitalised patients with Chronic Liver Disease, stratified by sex.

| Variables | SyNCA Upper Limbs^b^ | | | |
| --- | --- | --- | --- | --- |
|  | Female  n=12 | p-value | Male  n=35 | p-value |
| AMMI UL^a^ | -0.138 | 0.668 | -0.634 | **<0.001** |
| Variables | SyNCA Lower Limbs^c^ | | | |
|  | Female  n=12 | p-value | Male  n=35 | p-value |
| AMMI LL^a^ | -0.123 | 0.703 | -0.387 | **0.022** |

Legend: ^a^ Pearson's correlation coefficient; ^b^ SyNCA scores for the deltoid, biceps, triceps, thumb adductor, and dorsum of the hands (upper limbs); ^c^ SyNCA scores for the quadriceps, gastrocnemius, bony structures, and oedema (lower limbs). Abbreviations: AMMI: Appendicular Muscle Mass Index (kg/m²) obtained by Dual-energy X-ray Absorptiometry; UL: Upper Limbs; LL: Lower Limbs; SyNCA: Systematic Nutritional Clinical Assessment.
